# Effects of Blood Pressure and Sex on the Change of Wave Reflection: Evidence from Gaussian Fitting Method for Radial Artery Pressure Waveform

**DOI:** 10.1371/journal.pone.0112895

**Published:** 2014-11-10

**Authors:** Chengyu Liu, Lina Zhao, Changchun Liu

**Affiliations:** 1 School of Control Science and Engineering, Shandong University, Jinan, Shandong, China; 2 Institute of Cellular Medicine, Newcastle University, Newcastle upon Tyne, Tyne and Wear, United Kingdom; Max-Delbrück Center for Molecular Medicine (MDC), Germany

## Abstract

An early return of the reflected component in the arterial pulse has been recognized as an important indicator of cardiovascular risk. This study aimed to determine the effects of blood pressure and sex factor on the change of wave reflection using Gaussian fitting method. One hundred and ninety subjects were enrolled. They were classified into four blood pressure categories based on the systolic blood pressures (i.e., ≤110, 111–120, 121–130 and ≥131 mmHg). Each blood pressure category was also stratified for sex factor. Electrocardiogram (ECG) and radial artery pressure waveforms (RAPW) signals were recorded for each subject. Ten consecutive pulse episodes from the RAPW signal were extracted and normalized. Each normalized pulse episode was fitted by three Gaussian functions. Both the peak position and peak height of the first and second Gaussian functions, as well as the peak position interval and peak height ratio, were used as the evaluation indices of wave reflection. Two-way ANOVA results showed that with the increased blood pressure, the peak position of the second Gaussian significantly shorten (*P*<0.01), the peak height of the first Gaussian significantly decreased (*P*<0.01) and the peak height of the second Gaussian significantly increased (*P*<0.01), inducing the significantly decreased peak position interval and significantly increased peak height ratio (both *P*<0.01). Sex factor had no significant effect on all evaluation indices (all *P*>0.05). Moreover, the interaction between sex and blood pressure factors also had no significant effect on all evaluation indices (all *P*>0.05). These results showed that blood pressure has significant effect on the change of wave reflection when using the recently developed Gaussian fitting method, whereas sex has no significant effect. The results also suggested that the Gaussian fitting method could be used as a new approach for assessing the arterial wave reflection.

## Introduction

The shapes of artery pressure waveform are determined by the cardiac ejection function and the mechanical and geometric properties of the systemic arteries. Changes of the artery pressure waveform features have been accepted as the risk indicators of cardiovascular diseases [1], [2]. It is traditionally accepted that clinically measured artery pressure waveform contains both the forward and backward components [3]. Recent years, the backward component (i.e., wave reflection from the periphery to the heart, also named reflected component) has been recognized as an important indicator of cardiovascular risk [4]–[7]. However, the underlying physiological mechanisms of the forward and reflected components have not been fully understood. In healthy subjects, the reflected component normally returns to the central aorta in diastole and acts to maintain diastolic perfusion pressure in the coronary artery circulation. However, if the reflected component comes back earlier to the heart (usually in the late systole), it will conduce the rise of the central systolic and pulse pressures and will depress the coronary perfusion pressure [5]. For these reasons, it is important to accurately estimate the amount and location of the wave reflection [8]–[12].

O'ourke *et al* and Segers *et al* reported that wave reflection sites shift proximally toward the heart with the advancing age [13], [14]. On the contrary, Mitchell *et al* found that the wave reflection sites exhibit distal shifts with the advancing age due to the impedance fitting between the central aorta and proximal muscular arteries [15]. Sugawara *et al* used a combination of artery pressure waveform analyses and 3D MRI to locate the reflection sites and suggested that the major reflection sites did not change with aging until 65 years of age but shifted distally thereafter [5]. Therefore, it is controversial about the wave reflection sites. In addition, all of the above studies are mainly based on the analysis of the time-reference point features (e.g., the start, peak and inflection points) from the original pulse waveforms, or based on the analysis of the derived clinical indices, such as pulse wave velocity (PWV). However, the identification methods for the time-reference point features are optional and non-uniform, which could induce a failure to compare the results from the different researchers [1], [11], [12], [16]–[18]. Besides, the artery length measurement is very complicated when using a 3D MRI technique for PWV detection [19], which limited the practical applications. So the discrepancy in the above literatures is not surprising.

Recently, the model-based pulse decomposition methods have been used for the pulse forward and backward propagation analysis. In this case, the artery pressure waveform is often decomposed into several independent sub-waves using the different mathematic functions, such as the triangular [20], logarithmic normal [21], [22] and Gaussian functions [11], [12], [23]–[25]. Among them, pulse decomposition based on Gaussian fitting and particle swarm optimizer (PSO) algorithm could obtain both good fitting accuracy and fast calculation efficiency in our previous studies [11], [12]. In addition, to investigate the physiological relevance of the characteristic features from the modeled Gaussian functions, we compared them between the normal subjects and heart failure patients and found that significant changes were presented in the heart failure patients [26].

The previous studies have showed that the wave reflection changed with the age and hypertension factors when using the traditional clinical indices, such as pulse wave velocity (PWV), augmentation index (AI), reflection index (RI) and stiffness index (SI) [15], [27]–[29]. However, for the evaluation of wave reflection based on the modeling method, previous studies mainly paid attention to the effect of aging [13]–[15], [19]. In this study, we aimed to explore if there are changes of wave reflection with the blood pressure and sex differences in peripheral artery when using the Gaussian fitting method.

## Methods

### Ethics statement

One hundred and ninety subjects (105 men and 85 women; aged between 18 and 75) were enrolled at the Qilu Hospital of Shandong University. All subjects gave their written informed consents to participate in the study, and confirmed that they had not participated in any other ‘clinical trial’ within the previous three months. The study obtained a full approval from the Clinical Ethics Committee of the Qilu Hospitals of Shandong University and all clinical investigation was conducted according to the principles of expressed in the revised guidelines of Declaration of Helsinki – the fifth revision in the Edinburgh 2000 [30].

### Subjects

A comprehensive subject sample including the healthy volunteers (n = 57) and the cardiovascular disease subjects (n = 133) was included in this study. The cardiovascular disease subjects included: coronary heart disease (n = 53), congestive heart failure (n = 42) and dyslipidemia (n = 38). The coronary heart disease subjects should be consistent with one of the following symptoms: 1) The electrocardiogram (ECG) waves presented a typical myocardial infarction abnormality; or 2) A horizontal or oblique downward superior to 0.1 mV could be found on the ECG ST-segment. The congestive heart failure subjects should be consistent with one of the following symptoms: 1) Accord with the class II-III of the New York Heart Association (NYHA) Functional Classification; or 2) Left ventricular ejection fraction (LVEF) is lower than 50% with ultrasonic cardiogram (UCG) detection. The dyslipidemia subjects should have a higher low-density lipoprotein, total cholesterol or triglycerides. The healthy volunteers should have the normal performances in the UCG and ECG checks. Women who were pregnant or subjects with severe organ damage or psychiatric disorders had been excluded. No subjects had taken medications or smoked cigarettes before the test. All of the potential risks and procedures of the study were explained to the subjects, and they gave their written informed consent to participate in this study.

### Experimental protocol

**Figure 1:**
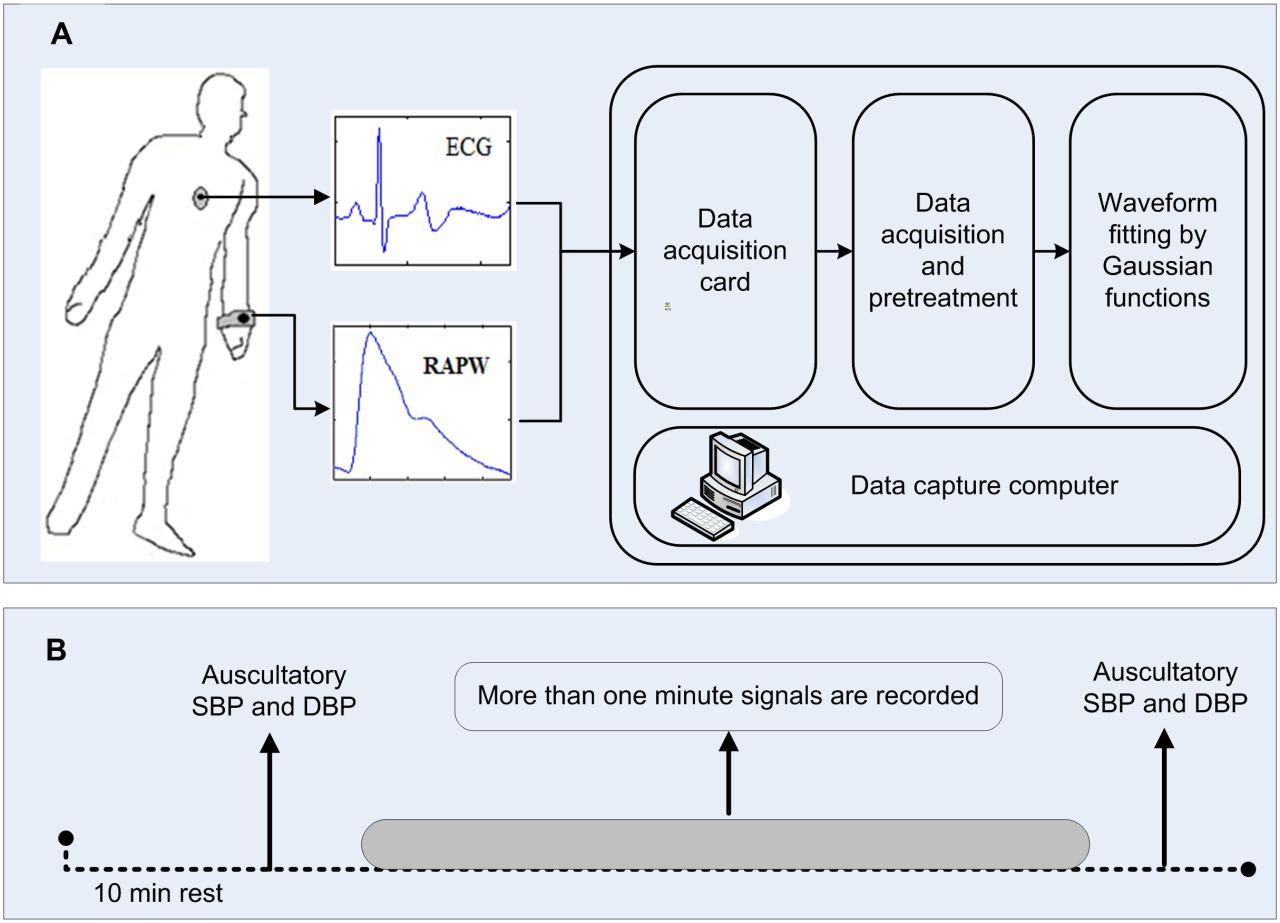
Schematic diagram of the measurement system and experimental procedure. (A) ECG and radial artery pressure waveforms (RAPW) signals were synchronously recorded with a sampling rate of 1000 Hz and were converted into digital signals using a 16-bit A/D data acquisition card. (B) Auscultatory systolic blood pressure (SBP) and diastolic blood pressure (DBP) were recorded manually at the beginning and end of the signal recording (more than one minute) for each subject.

Figure 1 gives a schematic diagram illustrating the measurement system and experimental procedure. All the measurements were undertaken in a quiet, temperature-controlled measurement room in the Qilu Hospital of Shandong University, which provided a stable temperature at the level of 25±3°C. Before the formal recording, each subject lay supine on a measurement bed for a 10 min rest period to allow cardiovascular stabilization. Three electrocardio-electrode clamps were attached to the right wrist, the right ankle and the left ankle respectively, to acquire the standard limb II-lead ECG signal. The piezoresistive sensor was attached to the left wrist to acquire the radial artery pressure waveforms (RAPW) signal. A cuff of mercurial sphygmomanometer was wrapped on the subject’s right brachia for the auscultatory blood pressures measurement manually, i.e., systolic blood pressure (SBP) and diastolic blood pressure (DBP). The procedure for blood pressure measurement was followed the guidelines recommended by the British Hypertension Society and American Heart Association [31], [32]. All the measurements were performed by an experienced operator to ensure the electrocardio-electrode clamps, the piezoresistive sensor and the cuff were attached well.

The standard limb II-lead ECG and RAPW signals were synchronously recorded with a sampling rate of 1000 Hz for more than 1 min and were converted into digital signals using a 16-bit A/D data acquisition card (National Instruments, USA). During the signal recording, the subjects were asked to keep the regular and gentle breathing. Auscultatory SBP and DBP were recorded manually at the beginning and end of the signal recording from the right brachia. The mean arterial pressure (MAP) and pulse pressure (PP) were calculated using the classic formula: MAP  =  DBP + (SBP−DBP)/3 and PP  =  SBP-DBP. For each subject, SBP, DBP, MAP and PP all used the mean value of the two measurements.

### Signals pretreatment

**Figure 2:**
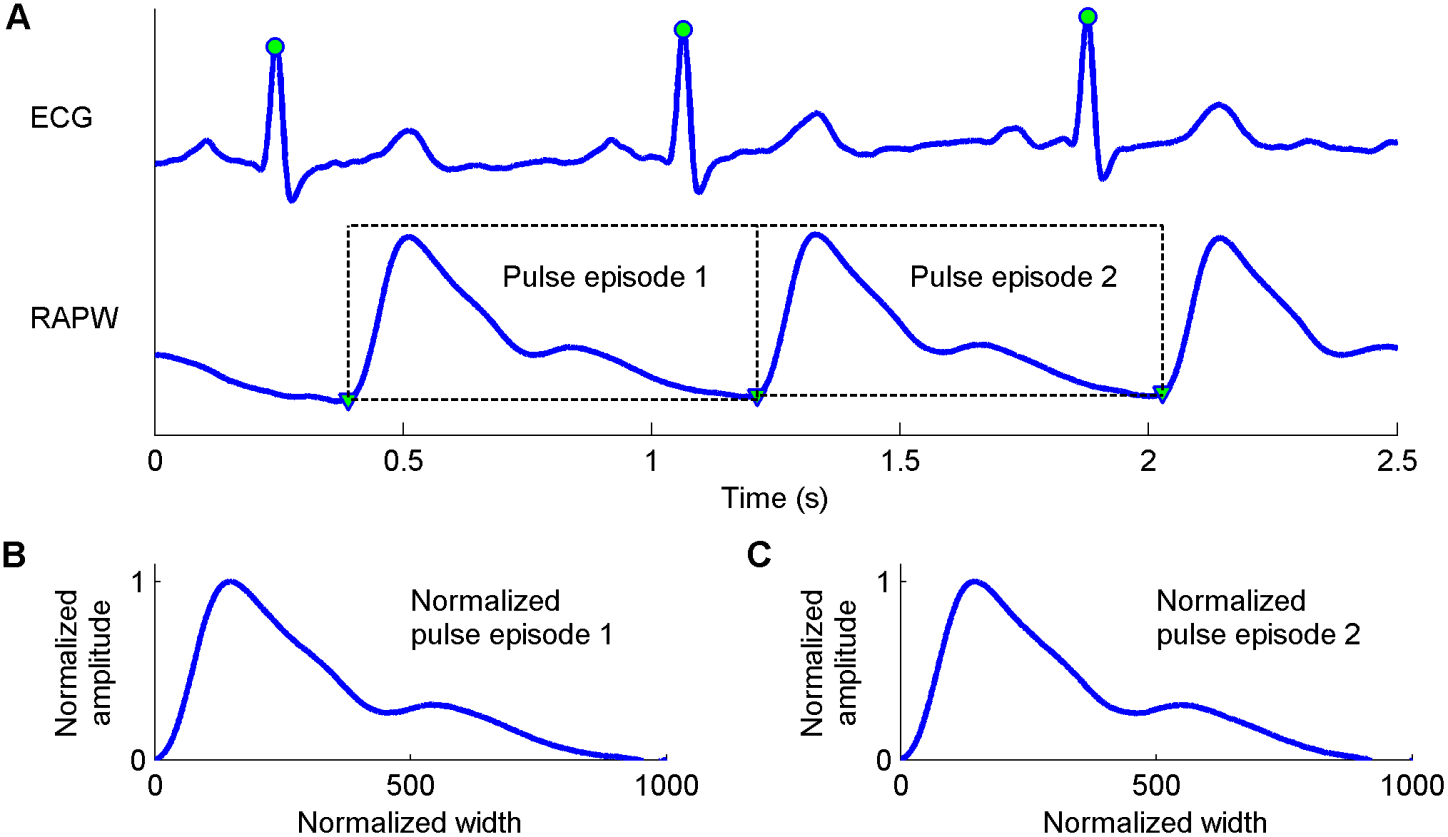
Demonstration examples of the ECG and RAPW signals and the construction process for the normalized pulse episode. (A) ECG and RAPW signals and their feature information: the detected R-wave peaks are denoted as “•”and the starting points of RAPW are denoted as “▾”, (B) and (C) the normalized pulse episodes corresponding to the original pulse episodes in sub-figure (A) with a width of 1000 points and amplitude to unity between 0 and 1.

Figure 2 (a) gives a demonstration of the synchronously recorded ECG and RAPW signals. First, the slow varying components (0–0.05 Hz) were removed from the ECG and RAPW signals. Second, the R-wave peaks of the ECG were detected using the Wavelet Transform Modulus Maxima method [33]. Ectopic beats were identified and excluded using our previously developed method [34]. After the location of R-wave peaks, the corresponding pulse feet (start of pulse) were found. Solà et al’s method was used to detect the pulse feet [17], which was based on the parametric modeling of the rising edge of a pulse waveform. The RAPW signal was then segmented between the starting points of two consecutive pulses. The first ten successive cardiac cycle pulse episodes without the ectopic beats were used for the subsequent analysis. Using ten pulse episodes could ensure the variation over a respiratory period was included.

Let 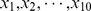 denotes the selected ten cardiac cycle pulse episodes from the RAPW signal. Each 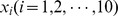 will be enlarged or shortened to 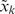, which has the uniform length of 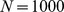 (i.e. the width normalization). After the width normalization, the amplitude normalization was executed using the following formula:
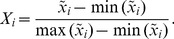
(1)After the amplitude normalization, each pulse episode 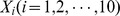 will have the same length 1000 and the same amplitude range between 0 and 1. The construction process for the normalized pulse episode of RAPW signal was shown in Figure 2. In Figure 2 (A), two pulse episodes were separated from the RAPW signal and the corresponding normalized pulse episodes were shown in Figure 2 (B) and (C)

### Waveform fitting method

Our previous study [12] reported that the radial pulses could be accurately and reliably modeled using three positive Gaussian functions. So three Gaussian functions were used here and they were denoted as *f*_1_(*n*), *f*_2_(*n*) and *f*_3_(*n*). Each Gaussian function *f_k_*(*n*) (*k* = 1, 2, 3) had 1000 points (*n* = 1, 2, …, 1000) and was determined by three sub-wave parameters: the peak height *H_k_*, half-width *W_k_* and peak position *C_k_*. The Gaussian functions are defined as follows:
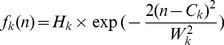
(2)where *n* = 1, 2, …, 1000, *k* = 1, 2, 3, and *C_k_* satisfies the following condition: 1<*C*_1_<*C*_2_<*C*_3_<1000.

**Figure 3:**
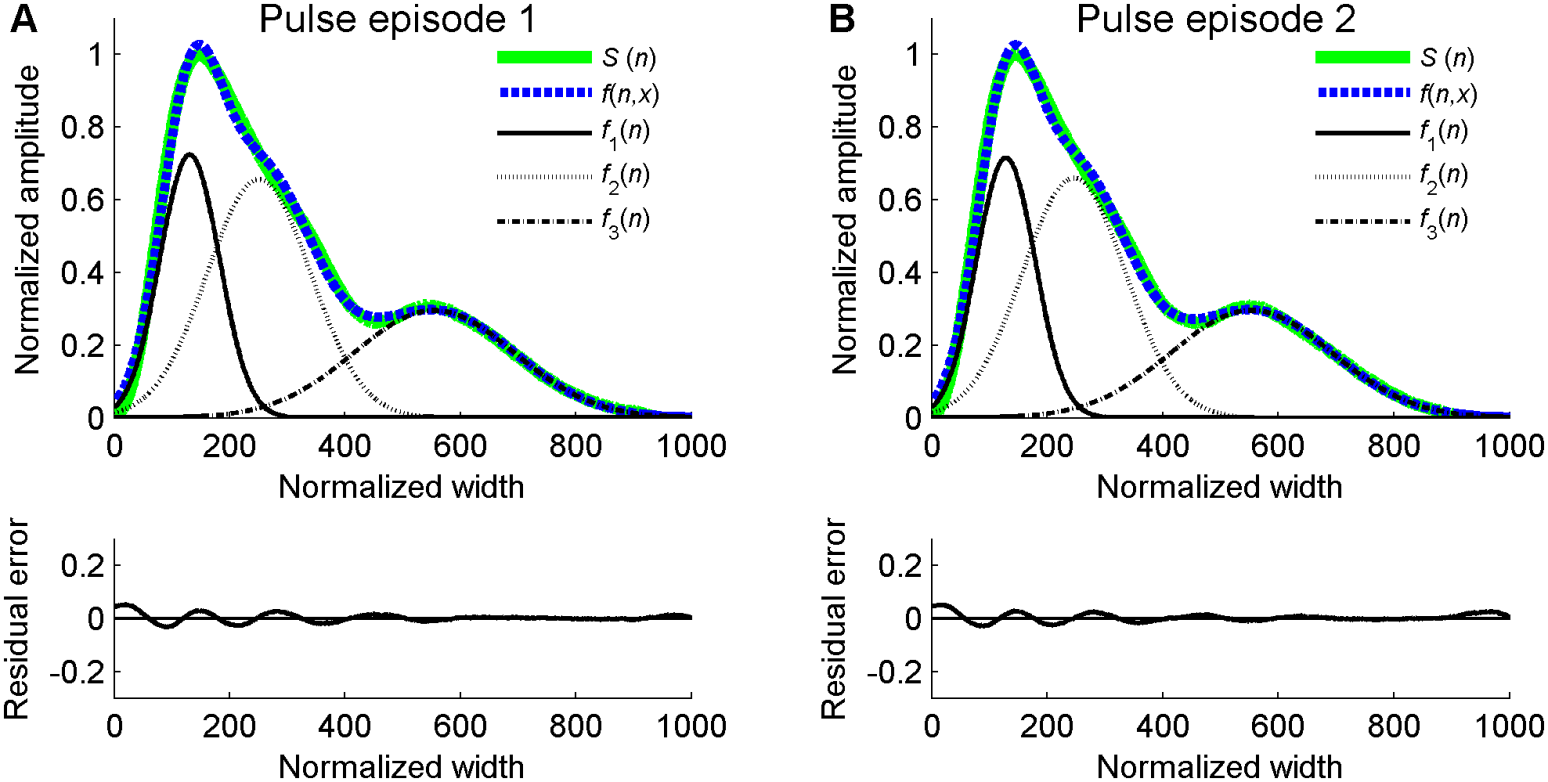
Examples of waveform fitting for the normalized pulse episodes. (A) Fitting results for the normalized pulse episode 1 in Figure 2 (B), (B) fitting results for the normalized pulse episode 2 in Figure 2 (C). In each sub-figure, the upper panel shows the original normalized pulse episode *S*(*n*), the fitting curve *f*(*n*,*x*) and the corresponding three Gaussian functions *f*_1_(*n*), *f*_2_(*n*) and *f*_3_(*n*) from left to right in turn. The bottom panel shows the corresponding residual error of the waveforms fitting.

After the nine parameters *H_k_*, *W_k_* and *C_k_* were determined, the superimposed curve *f*(*n*,*x*) of the three Gaussian functions was regarded as the fitted curve for the normalized pulse episode *S*(*n*):
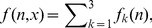
(3)where *x* = [*H_k_*, *W_k_*, *C_k_*] (*k* = 1, 2, 3) was the sub-wave parameter vector. The objective function for optimization can be defined as follows:



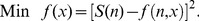
(4)We used our recently developed two-stage particle swarm optimizer (TSPSO) to achieve the parameter optimization in formula (4) and the detailed description about TSPSO could be found in [11]. Figure 3 showed the examples of waveform fitting for the normalized pulse episodes from Figure 2 (B) and (C).

### Evaluation indices for the change of wave reflection

For Gaussian fitting method, the first Gaussian is usually regarded as the forward component and the second Gaussian is usually regarded as the main reflected component [11], [12], [22], [24], [25]. So in this study, we analyzed both the peak position (*C*_1_ and *C*_2_) and peak height (*H*_1_ and *H*_2_) indices from the first and second Gaussian functions, as well as two derived indices: the peak position interval between these two Gaussian functions defined as *C*_ref-forw_  =  *C*_2_–*C*_1_ and the peak height ratio between them defined as *H*_ref/forw_  =  *H*_2_/*H*_1_. All six indices were calculated by averaging the results from the ten successive normalized pulse episodes.

### Statistical analysis

Two-way ANOVA was used to examine the effects of blood pressure and sex on the selected physiological variables and the evaluation indices. All of the data were reported as number (No.) or mean±standard deviation (SD). Statistical significance was set a priori at *P*<0.05. All statistical analysis was performed using the Statistical Package for Social Sciences (V19.0, IBM Corp. Released 2010, Armonk, New York, USA).

## Results

Table 1 shows the results of the selected physiological variables from all 190 subjects in four blood pressure categories based on the SBP values (i.e., ≤110, 111–120, 121–130 and ≥131 mmHg) as well as stratified for the sex factor. As expected, all the blood pressure variables (SBP, DBP, MAP and PP) have significant differences in the SBP category (all *P*<0.01) whereas they have no significant differences in the sex category (all *P*>0.05). Age and LVET variables have no significant differences in either the sex or SBP category (all *P*>0.05). The other four variables (height, body mass, body mass index and heart rate) have significant differences in the sex category (all *P*<0.05) but only body mass and body mass index have significant differences in the SBP category (both *P*<0.05). In addition, all variables have no significant differences for the interaction between the sex and SBP categories (all *P*>0.05).

**Table 1:** Results of the selected physiological variables from all 190 subjects.

Variables	Sex	SBP category, mmHg	ANOVA results		
		≤110	111–120	121–130	≥131	Sex	SPB	Interaction
No.	Men	22	29	33	21	NS	NS	NS
	Women	17	25	25	18			
Age, year	Men	47±12	50±14	51±11	49±7	NS	NS	NS
	Women	50±10	51±11	48±9	49±10			
Height, cm	Men	171±8	172±7	170±7	171±9	*P*<0.01	NS	NS
	Women	158±9	159±10	157±7	157±8			
Body mass, kg	Men	69±11	70±9	72±10	75±11	*P*<0.01	*P*<0.05	NS
	Women	51±7	51±8	55±11	59±10			
BMI, kg/m^2^	Men	23.6±2.8	23.7±2.9	24.9±3.1	25.6±3.0	*P*<0.01	*P*<0.01	NS
	Women	20.4±2.3	20.2±2.5	22.3±2.5	23.9±2.9			
HR, bpm	Men	67±11	67±10	66±9	69±11	*P*<0.05	NS	NS
	Women	70±9	69±11	71±10	73±8			
LVEF, %	Men	56±12	58±11	57±3	55±10	NS	NS	NS
	Women	57±13	60±10	59±11	56±11			
SBP, mmHg	Men	104±3	115±4	126±3	135±5	NS	*P*<0.01	NS
	Women	104±4	116±3	125±3	134±3			
DBP, mmHg	Men	67±8	73±7	77±6	82±7	NS	*P*<0.01	NS
	Women	66±6	72±8	75±7	80±6			
MAP, mmHg	Men	79±7	87±6	95±6	103±8	NS	*P*<0.01	NS
	Women	77±5	85±7	93±7	99±6			
PP, mmHg	Men	37±6	42±7	49±5	53±7	NS	*P*<0.01	NS
	Women	38±7	44±6	50±5	54±6			

Note: Data are expressed as number (No.) or mean±SD. BMI: body mass index; HR: heart rate; LVEF: left ventricular ejection fraction; SBP: systolic blood pressure; DBP: diastolic blood pressure; MAP: mean arterial pressure; PP: pulse pressure.

Table 2 shows the results of the evaluation indices from the first and second Gaussian functions. All six indices have no significant differences in the sex category (all *P*>0.05). However, all six indices have significant differences (all *P*<0.01) in the SBP category except for the peak position *C*_1_. In addition, all six indices have no significant differences for the interaction between the sex and SBP categories (all *P*>0.05).

**Table 2:** Results of the selected evaluation indices from all 190 subjects.

Indices	Sex	SBP category, mmHg	ANOVA results		
		≤110	111–120	121–130	≥131	Sex	SPB	Interaction
*C*_1_	Men	136±11	135±12	140±11	138±12	NS	NS	NS
	Women	142±9	135±11	137±11	134±10			
*C*_2_	Men	260±16	251±12	246±13	233±14	NS	*P*<0.01	NS
	Women	265±15	256±14	248±15	235±15			
*C*_ref-forw_	Men	124±16	116±17	106±11	95±12	NS	*P*<0.01	NS
	Women	123±15	121±18	111±17	101±15			
*H*_1_	Men	0.73±0.11	0.70±0.12	0.66±0.12	0.61±0.13	NS	*P*<0.01	NS
	Women	0.71±0.12	0.70±0.12	0.67±0.13	0.62±0.10			
*H*_2_	Men	0.67±0.10	0.71±0.09	0.73±0.11	0.77±0.10	NS	*P*<0.01	NS
	Women	0.67±0.11	0.70±0.11	0.72±0.09	0.75±0.11			
*H*_ref/forw_, %	Men	92±16	101±17	111±18	126±20	NS	*P*<0.01	NS
	Women	94±17	100±17	107±19	121±19			

Note: Data are expressed as mean±SD.

For both men and women subjects, with the increase of SBP, the peak position *C*_2_ significantly shorten (*P*<0.01), the peak height *H*_1_ significantly decreased (*P*<0.01) and the peak height *H*_2_ significantly increased (*P*<0.01), inducing the significantly decrease of the peak position interval *C*_ref-forw_ (*P*<0.01) and the significantly increase of the peak height ratio *H*_ref/forw_ (*P*<0.01).

## Discussion and Conclusion

The previous studies have showed that the wave reflection changed with the age [5], [14] and hypertension [1] factors. In the current study, we aimed to explore if the change of wave reflection with the increase of blood pressure could be observed using the recently developed Gaussian fitting method. The radial artery pressure waveform was modeled by three Gaussian functions. The peak position and peak height information of the first and second Gaussian functions, as well as their derived indices, were used to evaluate the effects of blood pressure and sex factors on the change of wave reflection. Two-way ANOVA results showed that all evaluation indices have no significant differences in the sex category but have significant differences in the SBP category except for the peak position of the first Gaussian, confirming the change of wave reflection with the increase of blood pressure when using Gaussian fitting method.

As traditionally accepted, the first Gaussian is more likely to be linked with the ejecting blood function of left ventricle and could be regarded as the forward component of arterial pulse [26]. The second Gaussian possibly associated with the main backward component of arterial pulse [12], [24], [25]. In this study, the peak position *C*_2_ became smaller with the increase of blood pressure, indicating that the main backward component of pulse occurs earlier. Meanwhile, with the increase of blood pressure, significantly lower of the peak height *H*_1_ and higher of the peak height *H*_2_ were found. The former shows the amplitude decline of the forward component, which can indicate the strength decline of the ejecting blood function of left ventricle. The latter shows that the amplitude increases of the backward component, which may be caused by the increase of artery stiffness. Similar results were also reported in [13], [14]. However, the peak position *C*_1_ had no significant change with the increase of blood pressure, showing that the stable occurrence of the forward component of arterial pulse. In addition, all evaluation indices have no significant differences in the sex category (all *P*>0.05), and also have no significant differences for the interaction between the sex and SBP categories (all *P*>0.05), verifying that the sex factor does not take a part in the change of wave reflection in the peripheral artery.

There are also several widely used clinical indices (PWV, AI, RI and SI) for the assessment of wave reflection in clinical practice [15], [27]–[29]. These indices are mainly based on the analysis of the time-reference point features and their responding amplitude features in the pulse waveform. So the accurate measurement for the time-reference point features is very important [13]. If the reflected components move forward, they will mix together with the forward component and this will induce that the peak of the original pulse waveform becomes obtuse. Moreover, the determination for the time-reference point features is much easily influenced by the noises. Therefore, any potential improvements for the accurate assessment of the time-reference point features are clinically important, and worth further investigation. The Gaussian fitting method is one option of the potential improvements.

One unique aspect of the current study is to use the TSPSO algorithm to achieve the accurate waveform fitting. We have reported that the TSPSO method could achieve a high fitting accuracy for the RAPW signal [11]. The high fitting accuracy could be observed from the demonstration examples in Figure 3, where the residual error of waveform fitting is restricted within a relative low level. It is also worth to note that the acquired three Gaussian functions for the two consecutive normalized pulse episodes in Figure 3 are very similar, showing the fine stability of the Gaussian fitting method. These results suggested that the Gaussian fitting method could be used as a new approach for assessing the arterial wave reflection and to explore the relationship between the Gaussian features and different physiological or pathological factors. To explore the clinical significance of the acquired indices from the Gaussian fitting method, the comprehensive comparisons between these indices and the clinical indices (PWV, AI, RI and SI) could be our future work.
